# The Impact of Immune Microenvironment on the Prognosis of Pancreatic Ductal Adenocarcinoma Based on Multi-Omics Analysis

**DOI:** 10.3389/fimmu.2021.769047

**Published:** 2021-10-28

**Authors:** Bing Yang, Mingyao Zhou, Yunzi Wu, Yuanyuan Ma, Qin Tan, Wei Yuan, Jie Ma

**Affiliations:** ^1^ Center of Biotherapy, Beijing Hospital, National Center of Gerontology, Institute of Geriatric Medicine, Chinese Academy of Medical Sciences, Beijing, China; ^2^ Peking University Fifth School of Clinical Medicine, Beijing, China; ^3^ Department of Colorectal Surgery, National Cancer Center/National Clinical Research Center for Cancer/Cancer Hospital, Chinese Academy of Medical Sciences and Peking Union Medical College, Beijing, China; ^4^ Department of Pancreatic and Gastric Surgery, National Cancer Center/National Clinical Research Center for Cancer/Cancer Hospital, Chinese Academy of Medical Sciences and Peking Union Medical College, Beijing, China; ^5^ State Key Laboratory of Molecular Oncology, National Cancer Center/National Clinical Research Center for Cancer/Cancer Hospital, Chinese Academy of Medical Sciences and Peking Union Medical College, Beijing, China

**Keywords:** PDAC, risk-score, prognosis prediction, molecular subtype, immunotherapy sensitivity

## Abstract

Pancreatic ductal adenocarcinoma (PDAC) is a malignant tumor characterized by rapid progression, early metastasis, high recurrence, and limited responsiveness to conventional therapies. The 5-year survival rate of PDAC is extremely low (<8%), which lacks effective prognostic evaluation indicators. In this study, we used xCell to analyze infiltrating immune cells in a tumor and through the univariate and multivariate Cox analyses screened out two prognosis-related immune cells, CD4^+^T_N_ and common lymphoid progenitor (CLP), which were used to construct a Cox model and figure out the risk-score. It was found that the constructed model could greatly improve the sensitivity of prognostic evaluation, that the higher the risk-score, the worse the prognosis. In addition, the risk-score could also identify molecular subtypes with poor prognosis and immunotherapy sensitivity. Through transcriptome and whole-exome sequencing analysis of PDAC dataset from The Cancer Genome Atlas (TCGA), it was found that copy number deletion and low expression of CCL19 might be crucial factors to affect the risk-score. Lastly, validation of the above findings was confirmed not only in Gene Expression Omnibus (GEO) datasets but also in our PDAC patient samples, Peking2020 cohort.

## Introduction

Pancreatic ductal adenocarcinoma (PDAC) is one of the most fatal malignancies, characterized by rapid progression, early metastasis, high recurrence, limited responsiveness to conventional therapies, and a low 5-year overall survival (OS) rate (<8%) (1). As with other cancers, PDAC is considered as a complex genetic disease. The prognostic evaluation of PDAC is based on the characteristics of the tumor itself, such as TP53 deletion and KRAS mutation (2, 3). Thus, only a handful have finally entered clinical applications. There is a growing realization that tumor microenvironment (TME) plays an important role in tumorigenesis and development, where infiltrating immune cells are indispensable factors affecting treatment and prognosis (4). Compared with tumor cells, immune cells as normal somatic cells in the tumor immune microenvironment have less mutation burden, have less heterogeneity within the tumor and among patients, and are easier to serve as a reliable and stable prognostic evaluation scale. For example, the prognosis of triple-negative (TN) breast cancer can be assessed by measuring the amount of lymphocytic infiltration, and the greatest survival benefit from each 10% increase in lymphocytic infiltrate can be derived (5), while high immune cell infiltration is negatively correlated with the prognosis of brain lower-grade glioma, glioblastoma multiforme, and uveal melanoma (6). These all imply that immune cell infiltration has different effects on the prognosis in different tumor backgrounds. For the prognosis of PDAC, there is no consensus on the impact of immune cell infiltration.

PDAC has always been considered as a “cold” tumor with extensive myeloid-derived suppressor cells (MDSCs; for example, tumor-associated macrophage and myeloid-derived suppressor cells) (7, 8) and dense stromal tissue, which further hinders the flow of immune cells and makes it less sensitive to immunotherapy. On the other hand, this natural barrier may be more conducive to the homeostasis of local unique immune microenvironment in PDAC, making the infiltrated immune cells not prone to large fluctuations in a short time, which increases possibility and stability to assess the prognosis of PDAC by the infiltration of immune cells. In support of these inspiring discoveries, there are quite a few researchers predicting the prognosis of PDAC by analyzing immune-related genes, lncRNA and miRNA. Nevertheless, it is infiltrating immune cells that perform the final function. The prognosis assessment method based on immune-related genes [such as 4-chemokine(9) and 15-gene immune, stromal, and proliferation gene signature strategies(10)] all rely on whether chemokines or their related receptors happen to be expressed on the surface of infiltrated immune cells or not. This indirect evaluation method limits the accuracy of its evaluation to a certain extent. Similarly, lncRNA (11) and miRNA (12) often have multi-target characteristics downstream that may perform different functions under different inflammation backgrounds and lack sufficient predictive stability. Recently, there has been a rise in the molecular subtype study of PDAC combining TME and transcriptome analysis, for example, basal-like and activated stromal subtype proposed by Moffitt et al. (13) and pure basal-like and stromal activated subtypes identified by Puleo’s group (14), which all suggest that the prognosis of patients is bad. On the other hand, the panel of genes applied for molecular subtype has not yet reached a consensus. All data are based on the transcriptome, which increases the cost of prediction strategy, and complex algorithms hinder its clinical application. Therefore, we urgently need to find a more innovative, simpler, and more economical evaluation model to measure the prognosis of PDAC based on infiltrating immune cells directly.

With the promotion and popularization of second-generation sequencing technology, we can obtain sequencing data from multiple omics and multiple database centers, and we have a more integrated and accurate understanding of the PDAC immune microenvironment landscape. xCell is a very popular R package, launched in 2017 by Aran’s group, to infer the proportion of cell types in bulk RNA samples (15, 16). In our study, we use the xCell algorithm to predict 64 types of non-tumor cell in PDAC. Among them, there are 36 kinds of immune cells, of which only two are related to prognosis, CD4^+^ naïve T cell (CD4^+^T_N_) and common lymphoid progenitor (CLP), where CD4^+^T_N_ was positively correlated with prognosis, while CLP was contrasting. Then we combined them to construct Cox model, which can significantly improve the sensitivity of prognostic evaluation. The risk-score obtained by the model is negatively correlated with the prognosis. The feasibility has also been further confirmed in the Gene Expression Omnibus (GEO) database and our clinical PDAC patients, Peking2020 cohort. Lastly, by combining transcriptome and whole-exome sequencing analysis of PDAC dataset from The Cancer Genome Atlas (TCGA), it is found that CCL19 may be a crucial factor affecting the model.

## Materials and Methods

### Datasets and Clinical Information

PDAC level 3 expression profiles of RNA sequencing data [fragments per kilobase of transcript per million mapped reads (FPKM)], somatic mutation, copy number variation (CNV) data, and the corresponding clinical information of 143 PDAC patients were downloaded from TCGA (https://tcga-data.nci.nih.gov/tcga/). The patients who lack survival time or who had survival time of less than 30 days were removed, and only 136 PDACs were finally enrolled. Validation cohort, i.e., GSE71729, GSE102238, and GSE57495, was extracted from GEO datasets (https://www.ncbi.nlm.nih.gov/gds/). The gene expression information of normal pancreatic tissue (FPKM) was derived from Genotype-Tissue Expression (GTEx) *via* University of California, Santa Cruz, Xena (https://xenabrowser.net/datapages/?cohort=GTEX). All RNA sequencing data were normalized and transformed into log2(FPKM + 1); then unexpressed or extremely low-expressed genes in most of the samples (average log2(FPKM + 1) < 0.01) were filtered out. Matched clinical validation cohort, namely, Peking2020 Cohort, with 40 cases in total, originated from Cancer Center/National Clinical Research Center for Cancer/Cancer Hospital, wherein written informed consent was obtained from all patients (**Table 1**).

**Table 1:** Demographic characteristics of TCGA-PDAC and Peking2020 cohort.

Demographic characteristics		TCGA-PDAC	Peking 2020 cohort
Total case		136	40
Age		64.36 ± 10.95	60.93 ± 11.72
Gender	Male	71	26
	Female	65	14
Anatomic site of pancreas	Head	103	15
	Body	10	3
	Tail	8	14
	Other	15	8
Neoplasm histologic grade	High differentiation	16	2
	Medium differentiation	80	11
	Low differentiation	39	23
	Other	1	4
Pathologic_N	N0	35	14
	N1	100	19
	Other	1	7
Pathologic_T	T1	4	0
	T2	13	6
	T3	115	20
	T4	3	7
	Other	1	7
Tumor stage	I	11	4
	II	118	20
	III	3	12
	IV	3	1
	Other	1	3

“Other” represents unknown or missing information.

TCGA, The Cancer Genome Atlas; PDAC, pancreatic ductal adenocarcinoma.

### Estimation of Tumor-Infiltrating Immune Cells

The xCell online tool (https://xcell.ucsf.edu/) was used to perform enrichment analysis of tumor-infiltrating immune cell (TIIC) level, Immune Score, Stroma Score, and Microenvironment score based on gene expression data. The data analysis results of TCGA database were downloaded directly from the xCell portal website, while the GEO data needed to use the “sva” package in the R software 4.0.0 to remove the batch differences, and then we uploaded the standardized data to the xCell website for analysis. According to the median of the level of TIIC, 136 cases of PDAC patients in TCGA were divided into the high and low TIIC groups. Survival analysis in R software 4.0.0 was used to determine the cell types with prognostic value, and then the GEO datasets and Peking2020 Cohort were used for verification. Immunotherapy sensitivity was predicted through the ImmuneCellAI online perform (http://bioinfo.life.hust.edu.cn/ImmuCellAI#!/) based on gene expression data.

### Development of a Risk-Score

The risk-score of each patient was calculated based on the survival-related level of TIIC multiplied by the multivariate Cox regression coefficient (see the following formula for details), and the patients were divided into the high risk-score group and low risk-score group based on the median value of risk-score. The Kaplan–Meier (KM) survival analysis was performed on the two groups using log-rank test; receiver operating characteristic (ROC) curves of 1-year, 2-year, and 3-year survival prediction were drawn; then the area under the ROC curve (AUC) value was calculated.


$$risk-score=\Sigma \beta i*TIIC_{i}$$

where *βi* was the coefficient of the ith the survival-related TIIC, i.e., *β*(CD4^+^T_N_) = −0.16, *β*(CLP) = 0.63. *TIICi* represents the level of the ith survival-related TIIC.

### Analysis of Differentially Expressed Genes

Differentially expressed genes (DEGs) of the high risk-score group and low risk-score group were analyzed by R software 4.0.0 “edgeR” package. The screening threshold was set to log2(fold change) >1 and false discovery rate (FDR) <0.05, and then we used “pheatmap” package and “plot” package in R software 4.0.0 to draw heatmaps and volcano maps, respectively.

### Differentially Expressed Gene Function Enrichment Analysis

“Clusterprofiler” package in the R software 4.0.0 was used to perform functional enrichment analysis on DEGs, including Gene Ontology (GO) analysis and Kyoto Encyclopedia of Genes and Genomes (KEGG) pathway enrichment analysis, where GO analysis included molecular function (MF), biological process (BP), and cellular component (CC) analyses.

### Protein–Protein Interaction Network

Through Search Tool for the Retrieval of Interacting proteins (STRING, https://string-db.org) database, the protein–protein interaction (PPI) network of DEGs, namely, PPI, was established. The comprehensive score>0.4 was used as the criterion for the existence of interaction. Then Cytoscape software was used to rebuild the PPI network, and the Cytoscape MCODE plug-in was used to find core clusters that located densely connected areas and calculated the connectivity of network nodes.

### Tumor Mutation Burden Analysis

Tumor mutation burden (TMB) was defined as the total number of somatic mutations (including single-nucleotide variants (SNVs), missense mutation, and insertion–deletion (INDELs) per million bases in the coding region of exons). Somatic mutation data were analyzed using VarScan2, and then TMB was calculated as the total number of somatic mutations/all bases, and the unit was mutations/Mb. In this study, the “Maftools” package in R software 4.0.0 was used to calculate the TMB of each sample.

### Copy Number Variation Analysis

The copy number information of PDAC from TCGA was annotated according to the position information of grch38 genome; set the normal copy change as 0, then single copy amplification as 1, double copy even multiple copy amplification as 2, single copy deletion as −1, and double copy or multiple copy deletion as −2. Then, the chi-square test was used to select the differential copy number between the two groups in the high-risk group and low-risk groups (p < 0.05); the Kruskal test was used to determine the copy number differential genes related to gene expression; and the Gene Set Enrichment Analysis (GSEA) was used for functional analysis.

### Multiplex Immunofluorescence Staining

Multiplex immunofluorescence staining was performed according to a sequential multiplexed immunofluorescence protocol (17). Briefly, co-stain CD3 (Cat #ab16669, RIDD: AB_443425, Abcam), CD4 (Cat #ab133616, RIDD: AB_2750883, Abcam) and CD45RA (Cat #ab755, RIDD: AB_305970, Abcam), or CD127 (Cat #DF6362, RIDD: AB_2838326, Affinity), CD135 (Cat #DF8546, RIDD: AB_2841750, Affinity), and CCL19 (Cat #13397-1-AP, RIDD: AB_2071529, ProteinTech). Then, corresponding secondary antibodies used were fluorescein isothiocyanate–Tyramide Signal Amplification (FITC-TSA) (PPD520, Panovue) for CD3 or CCL19 detection, CY3-TSA (PPD570, Panovue) for CD4 or CD127 detection, and CY5-TSA (PPD620, Panovue) for CD45RA or CD135 detection. Nuclei were highlighted using DAPI (D9542, Sigma-Aldrich). Finally, using Phenochart software (Version 1.08, PerkinElmer) and inForm image analysis software (Version 2.4, PerkinElmer), we estimated the number of co-located cells of CD3, CD4, and CD45RA per ×100 magnification field as CD4^+^T_N_ level (randomly select three fields at least). Similarly, the number of co-located cells of CD127 and CD135 was calculated as CLP level. For the scoring of CCL19, we first evaluated the staining intensity of whole tumor tissue at low magnification. Samples with no staining were scored 0, weakly stained samples scored 1, mildly stained samples scored 2, and strongly stained samples scored 3. We also calculated the number of positive cells from at least three high magnification fields chosen at random as well as their mean intensities. As described above, samples with <25% positive expression were scored 1, samples within the expression range of 25%–50% scored 2, samples within the expression range of 50%–75% scored 3, and samples with expression ≥75% scored 4. The final CCL19 expression was determined by multiplying the intensity score with the positive expression value.

### Statistical Analysis

Statistical analysis was performed using GraphPad Prism 8.0 and R software 4.0.0. Measurement data were expressed as mean ± standard deviation, using Student’s t-test. Counting data were expressed as percentage (%), using chi-square test. Survival curve was drawn by the KM method, using log-rank test. The correlation between immune cells was evaluated by Spearman’s correlation coefficient. p < 0.05 or FDR < 0.05 was considered statistically significant in all tests.

## Results

### A Cox Model Constructed by Combining the Intratumoral Infiltration of CD4^+^T_N_ and Common Lymphoid Progenitor Can Improve the Sensitivity of Predicting Overall Survival in The Cancer Genome Atlas Pancreatic Ductal Adenocarcinoma Discovery Cohort

**Figure 1:**
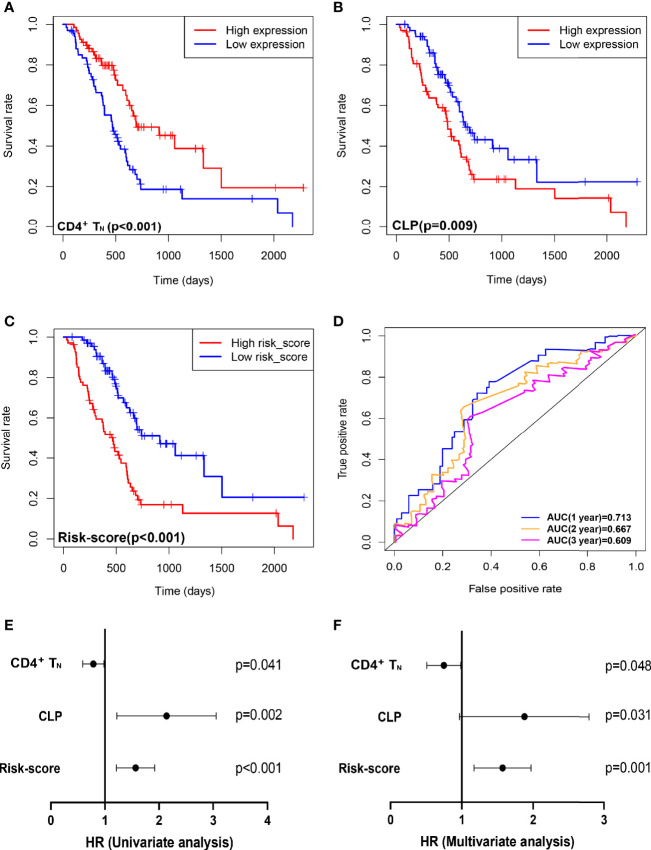
Combining intratumoral infiltrating CD4^+^T_N_ and CLP to construct a Cox model can improve the sensitivity of predicting OS in TCGA PDAC discovery cohort. **(A–C)** OS in CD4^+^T_N_, CLP, and risk-score high *vs*. low infiltrating cell/risk-score patients depicted by KM plots in TCGA PDAC discovery, respectively. **(D)** ROC curves to depict the accuracy of risk score in identifying poor OS in TCGA PDAC discovery cohort for 1, 2, and 3 years, respectively. **(E, F)** Forest plot showing the HR of CD4^+^T_N_, CLP, and risk score in the univariate and multivariate Cox analyses, respectively. OS, overall survival; KM, Kaplan–Meier; HR, hazard ratios; CLP, common lymphoid progenitor; TCGA, The Cancer Genome Atlas; PDAC, pancreatic ductal adenocarcinoma; ROC, receiver operating characteristic.

Based on the xCell algorithm, the type and level of immune cell infiltration in 136 PDAC cases were evaluated, and a total of 36 immune cells were predicted. Then KM survival analysis showed that only CD4^+^ naïve T cell (CD4^+^T_N_), CLP, and CD4^+^Th2 cell (Th2) were closely related to survival, where CD4^+^T_N_ was positively correlated with survival (p < 0.001, **Figure 1A**), while CLP and CD4^+^Th2 cell were negatively correlated with survival (p = 0.009, **Figure 1B**; p = 0.022, **Supplementary Figure S1A**). Furthermore, combined with clinical factors for univariate and multivariate analyses, only CD4^+^T_N_ and CLP could be regarded as independent risk factors, but the p-value was only slightly significant (**Figures 1E, F** and **Table 2**). However, when combining CD4^+^T_N_ and CLP to construct a proportional hazards model (Cox model), its clinical significance could be dramatically improved (p < 0.001, **Table 2**). Based on the individual point of risk-score calculated through the Cox model, PDAC patients were divided into the low risk-score and high risk-score groups (median set as cutoff). KM curves confirmed that the high risk-score group had much worse prognosis than the low risk-score group (p < 0.001, **Figure 1C**). Additionally, the risk-score could estimate OS of patients much sensitively, as the AUC was 0.713, 0.667, and 0.609 for 1-year, 2-year, and 3-year OS, respectively, and the discrimination index (C-index) was 0.706 (**Figure 1D**). Furthermore, this result was also verified in GSE71729 dataset (p = 0.027, **Supplementary Figures S1B, C**).

**Table 2:** Univariate and multivariate analyses for CD4^+^T_N_, CLP, and risk score.

Variables	Univariate analysis			Multivariate analysis		
	HR	95% CI of HR	p-Value	HR	95% CI of HR	p-Value
Age	1.02	0.995–1.045	0.118	1.02	0.993–1.049	0.154
Gender	0.772	0.457–1.306	0.335	0.886	0.477–1.646	0.701
Race	1.594	0.824–3.082	0.166	1.375	0.578–3.271	0.471
History of smoke	0.892	0.745–1.067	0.211	0.924	0.737–1.157	0.489
History of alcohol	1.031	0.577–1.841	0.919	1.192	0.622–2.285	0.596
History of diabetes	0.869	0.466–1.622	0.659	1.011	0.515–1.985	0.975
History of chronic pancreatitis	1.147	0.518–2.540	0.735	1.804	0.742–4.384	0.193
Prior malignancy diagnoses	0.98	0.302–3.186	0.974	0.589	0.169–2.049	0.405
Anatomic site of tumorigenesis	0.943	0.733–1.214	0.648	1.15	0.856–1.544	0.354
Neoplasm histologic grade	1.232	0.813–1.865	0.325	1.434	0.895–2.296	0.134
Pathologic_N	1.2	0.634–2.274	0.575	0.905	0.414–1.982	0.803
Pathologic_T	1.385	0.767–2.502	0.28	0.856	0.302–2.426	0.77
Tumor stage	1.523	0.673–3.449	0.313	1.343	0.312–5.784	0.693
T cell CD4^+^ naïve	0.77	0.598–0.989	0.041^*^	0.723	0.524–0.997	0.048^*^
Common lymphoid progenitor (CLP)	2.007	1.294–3.112	0.002^*^	1.732	1.050–2.855	0.031^*^
Risk_score	1.539	1.226–1.932	<0.001^***^	1.538	1.190–1.988	0.001^**^

For all panels, *p < 0.05, **p < 0.01, ***p < 0.001. The bold values: p < 0.05.

### Performance of the Risk-Score to Identify Molecular Subtypes and Immunotherapy Sensitivity

**Figure 2:**
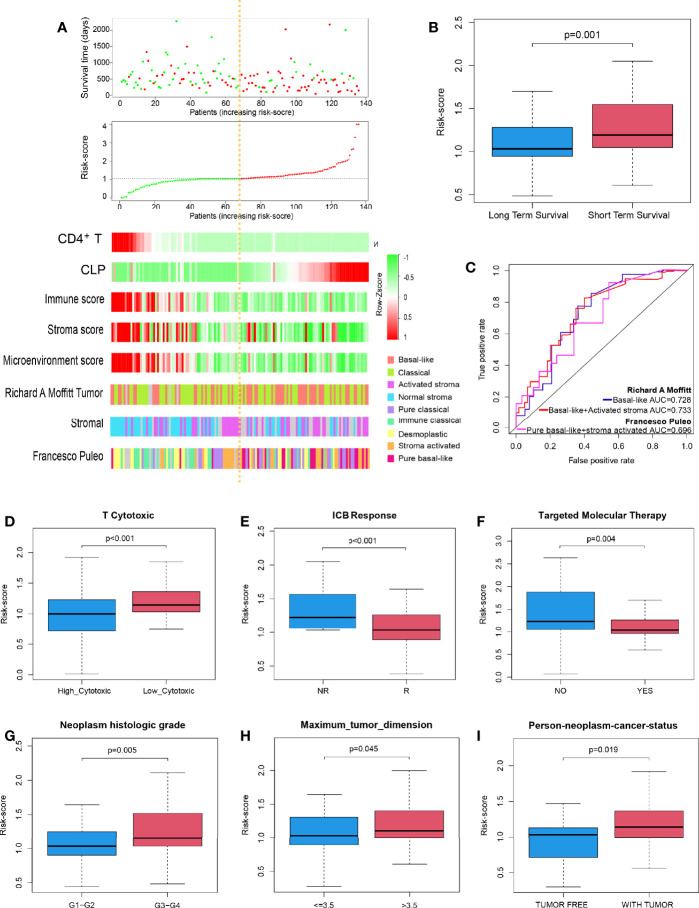
Performance of the risk-score to identify PDAC molecular subtypes with poor prognosis and immunotherapy sensitivity in TCGA discovery cohort. **(A)** Dot plot of survival. Vertical and horizontal axes respectively represent survival time and OS samples, ranked by increasing risk-score. Red and green colors represent dead and living OS cases, respectively. The second figure from the top is a dot plot of risk-score. Vertical and horizontal axes respectively represent risk-score and OS samples, ranked by increasing risk-score. Red and green colors represent high and low risk cases, respectively. The remaining figure is a heatmap for the level of CD4^+^T_N_, CLP infiltration, Immune Score, Stroma Score, Microenvironment score, and Moffit’s and Puleo’s molecular subtypes, ranked by increasing risk-score. **(B)** Box plot to depict risk-score between long-term survival and short-term survival. **(C)** ROC curves to depict the accuracy of risk-score in identifying PDAC molecular subtypes with poor prognosis in TCGA discovery cohort. **(D–I)** Box plot depicts risk-score of high T-cell cytotoxicity *vs*. low T-cell cytotoxicity, ICB no response *vs*. ICB response, no apply targeted molecular therapy *vs*. apply targeted molecular therapy, neoplasm histologic grade G1–G2 *vs*. G3–G4, tumor dimension ≤ 3.5 *vs*. > 3.5 cm, and person neoplasm cancer statue tumor free *vs*. with tumor, respectively. ICB, immune-checkpoint blockade; NR, no response; R, response; PDAC, pancreatic ductal adenocarcinoma; TCGA, The Cancer Genome Atlas; OS, overall survival; CLP, common lymphoid progenitor; ROC, receiver operating characteristic.

In succession, through the scatter plot of patient risk assessment, we found that risk-score was positively correlated with CLP level and negatively correlated with CD4^+^T_N_ level (**Figure 2A**). Meanwhile, with the increase of risk-score, the survival time of patients was gradually shortened, and the death events increased. Consistent with that, the risk-score was significantly higher in the short-term survival than the long-term survival (**Figure 2B**, p = 0.01). Interestingly, this risk-score also happened to be negatively correlated with the Immune Score, Stroma Score, and Microenvironment score. Previous studies had shown that molecular classification of pancreatic cancer was closely related to prognosis, but its complex algorithm has also become one of the important factors limiting its clinical application. Therefore, we were trying to explore whether the risk-score of our model could well identify high-risk molecular subtypes and replace its prognostic evaluation value. According to Moffitt’s algorithm (13) for molecular classification of PDAC, we found that with the increase of the risk-score, the proportion of basal-like and activated stroma corresponding to the molecular subtype with the worst prognosis increased. Also, through ROC curve analysis, it was found that Cox model was also highly sensitive to the prediction of basal-like molecular subtype (AUC = 0.728, **Figure 2C**). Furthermore, it was more sensitive to predict the joint basal-like and activated stroma molecular subtype (AUC = 0.733, **Figure 2C**). In addition, we also performed molecular classification of PDAC according to Puleo’s algorithm (14), and the results were consistent with those of the former: with the increase of risk-score, the proportion of pure basal-like and stroma-activated molecular subtypes associated with the worst prognosis increased as well as high prediction sensitivity (AUC = 0.696, **Figure 2C**).

Further studies suggested that risk-score could well predict the cytotoxicity of T cells in the microenvironment of PDAC and the sensitivity of PDAC patients to immune checkpoint inhibitor. The risk-score of the high cytotoxicity group and immunotherapy sensitive group was lower (p < 0.001 and p < 0.001, respectively; **Figures 2D, E**). Moreover, PDAC patients in whom molecular targeted therapy is applicable had a lower risk-score value (p = 0.004, **Figure 2F**). Besides that, the risk-score was positively correlated with the degree of dysplasia and tumor size; that is, the higher the degree of dysplasia, or the larger the tumor size, the higher the risk-score (p = 0.005, p = 0.045, **Figures 2G, H**). Meanwhile, patients with higher risk-score usually survived with tumors, and surgery to remove the tumor totally was more difficult (p = 0.019, **Figure 2I**).

### Risk-Score for Predicting Overall Survival Was Validated in Clinical Pancreatic Ductal Adenocarcinoma Patients

**Figure 3:**
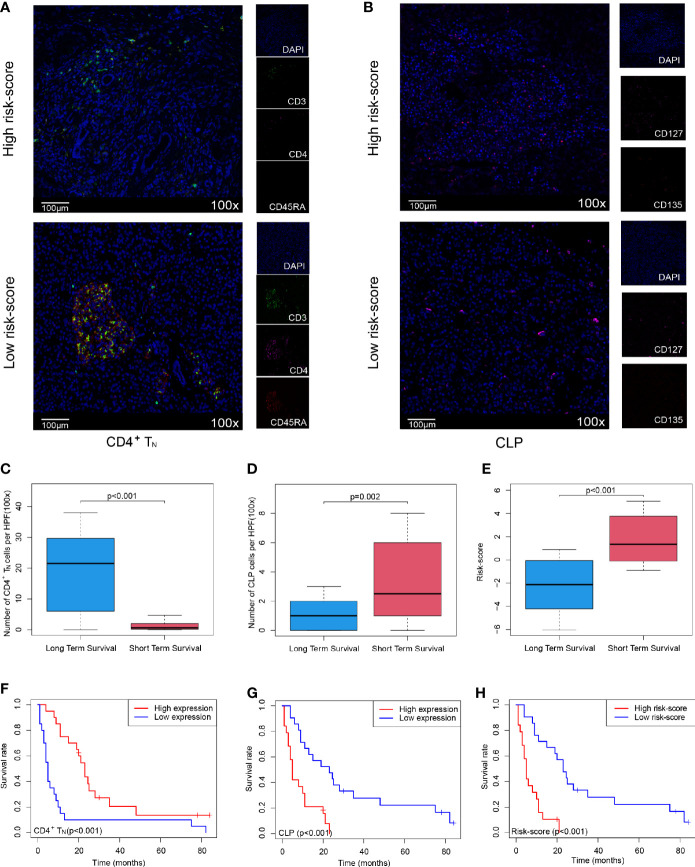
Validating the Cox model can improve the sensitivity of predicting OS in Peking2020 cohort. **(A, B)** Multiplex immunofluorescence analyses on CD4^+^T_N_ and CLP intratumoral infiltration in Peking2020 cohort (n = 40). CD4^+^T_N_ depicts co-localization staining for CD3^+^, CD4^+^, and CD45RA^+^. CLP depicts co-localization staining for CD127^+^ and CD135^+^ (randomly select three fields at least). **(C–E)** Box plot depicts the level of CD4^+^T_N_, CLP, and risk-score between long-term survival and short-term survival. **(F–H)** OS in CD4^+^T_N_, CLP, and risk-score high *vs*. low-infiltrating cell/risk-score patients depicted by KM plots in Peking2020 cohort respectively. OS, overall survival; KM, Kaplan–Meier; CLP, common lymphoid progenitor.

In order to further verify that the Cox model we constructed could indeed assess the prognosis of PDAC patients, we selected 40 PDAC patient samples, Peking2020 cohort, and followed the above algorithm to analyze the level of intratumoral infiltration of CD4^+^T_N_ (CD3^+^, CD4^+^, and CD45RA^+^) and CLP (CD127^+^ and CD135^+^) to calculate the risk-score of each case by multiplex immunofluorescence staining. As expected, the results of Peking2020 cohort were consistent with the above results. Contrary to the PDAC with high risk-score, the level of CD4^+^T_N_ was higher in the low risk-score group (**Figure 3A**), while the level of CLP tended to be lower (**Figure 3B**). Also, compared with the short-term survival group, the level of CD4^+^T_N_ in the long-term survival group was also higher (**Figure 3C**), while the level of CLP and risk-score was in reverse (**Figures 3D, E**). Furthermore, we conducted KM survival analysis and found that CD4^+^T_N_ was positively correlated with PDAC patient survival (p < 0.001, **Figure 3F**), but CLP was negatively correlated with patient survival (p < 0.001, **Figure 3G**), and after the joint analysis, the significance of the negative correlation between risk-score and prognosis was further confirmed (p < 0.001, **Figure 3H**). In addition, the same as the results of PDAC dataset from TCGA, the larger the tumor volume, the higher the risk-score (p < 0.001, **Supplementary Figure S1D**). Interestingly, PDAC in the tail of the pancreas had a higher risk-score than the head of the pancreas (p = 0.0316, **Supplementary Figure S1E**).

### Gene Expression and Function Profiled in High and Low Risk-Score Groups

**Figure 4:**
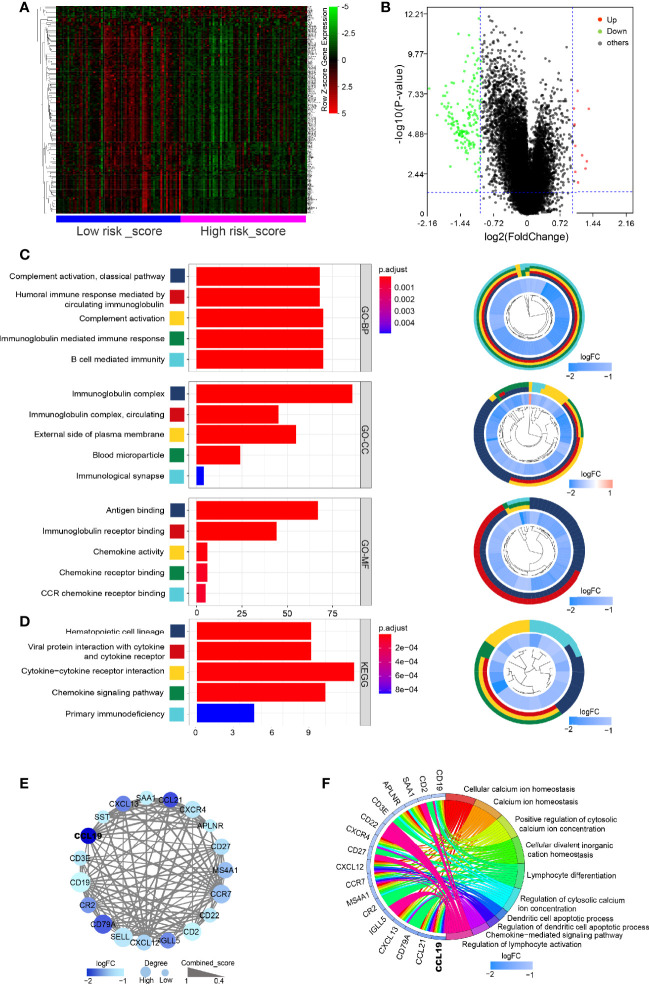
Gene expression and function profiled in high and low risk-score groups. **(A)** Heatmap of DEGs between low risk-score and high risk-score groups. Horizontal and vertical axes represent PDAC samples and genes, respectively. Genes with higher, lower, and same expression levels are shown in red, green, and black, respectively. Color bars on bottom of the heatmap represent sample types, with blue and pink indicating low and high risk-score samples, respectively. **(B)** Volcano plot demonstrating the enriched genes between low risk-score and high risk-score groups. Genes expression increased in high or low risk score is shown in red or green dots, respectively (log2(fold change) > 1 and FDR < 0.05). **(C, D)** Bar plot for the top 5 of BP-GO terms, CC-GO terms, MF-GO terms, and KEGG analysis of DEGs. Shown on the left is cir-plot for the correlation between DEGs and GO or KEGG terms. **(E)** PPI network of core DEGs by MCODE algorithm. **(F)** The correlation between core DEGs and top 5 BP-GO terms. DEGs, differentially expressed genes; FDR, false discovery rate; GO, Gene Ontology; BP, biological processes; CC, cellular component; MF, molecular function; KEGG, Kyoto Encyclopedia of Genes and Genomes; PPI, protein–protein interaction.

The above studies confirmed the role of risk-score in the prognostic evaluation of PDAC patients, so we tried to explore its potential molecular mechanism. First of all, principal component analysis (PCA) was conducted on the transcriptome data of healthy pancreas (combined with pancreas tissue from GTEx and paracancerous tissue of PDAC dataset from TCGA) and tumor tissue (PDAC dataset from TCGA). The results showed that the mRNA expression was significantly different between normal tissues and cancer tissues, while there was no significant difference between the low risk-score and high risk-score groups (**Supplementary Figures S1F, G**). However, through the analysis of heatmaps and volcano maps, we found that there were still 172 DEGs between the low risk-score and high risk-score groups, among which 162 genes were downregulated and 10 genes were upregulated in the high risk-score group (**Figures 4A, B**). Then, GO and KEGG analysis of these 172 DEGs revealed that they were mainly concentrated in immune-related functions and pathways, for example, Complement activation by classical pathway, Immunoglobulin-mediated immune response, and Cytokine–cytokine receptor interaction (**Figures 4C, D**). Furthermore, the 172 DEGs were analyzed for PPI. Then, the most important core interaction protein network was screened out through MCODE algorithm. It was found that CCL19 occupied one of the most core positions and interacted closely with other genes (**Figure 4E**). Moreover, the function of core interaction protein was mainly related to regulation of lymphocyte activation and differentiation, chemokine-mediated signaling pathway, etc. (**Figure 4F**).

### Whole-Exome Sequencing Data Showed the Difference Between High and Low Risk-Score Groups

**Figure 5:**
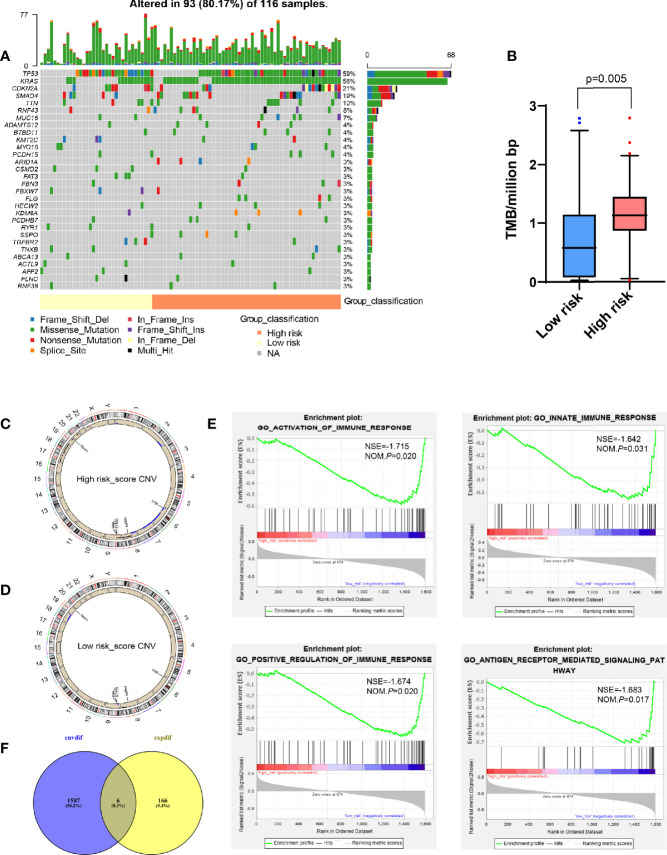
Analysis of whole-exome sequencing data showed the difference between high and low risk-score groups. **(A)** Oncoplots for TMB analysis between high and low risk-score groups, showing the top 30 mutated genes. The upper barplot indicates the number of mutations per sample, while the right barplot shows the number of mutations per gene. The mutation types were added as annotations at the bottom. **(B)** Box plot to show the TMB between high and low risk-score groups. **(C, D)** Circos plot for CNV in high and low risk-score groups, respectively. The outermost layer is the chromosome model, and the next two layers illustrate the CNV (single copy amplification is shown in black dots, double even multiple copy amplification is in red dots, and deletion is in blue dots). **(E)** Venn diagram analysis between the different CNV and DEGs from transcriptome. **(F)** GSEA for different CNVs. The vertical axis represents enrichment score. The enrichment score increased with the number of enriched genes and vice versa. CNV, copy number variation; GSEA, Gene Set Enrichment Analysis; ES, Enrichment Score; NES, Normalized Enrichment Score; TMB, tumor mutation burden; DEGs, differentially expressed genes.

Since previous studies had shown that TMB could affect tumor immune infiltration (18, 19), we further analyzed the TMB in PDAC. It was found that PDAC was a “cold” tumor with low TMB as reported in the literature (2, 3), but compared with the low risk-score, the high risk-score group still has higher TMB, such as conventional TP53 and KRAS mutation (**Figures 5A, B**), However, these mutations did not make the corresponding gene expression different at the transcriptome level, so it did not affect the expression of DEGs obtained by transcriptome analysis.

Compared with TMB, CNV between patients was more constant, and the frequency of variation was higher, which was one of the key events leading to tumor development (20–22). However, there were fewer studies on the tumor immune microenvironment. Therefore, we tried to find the main CNV that could affect the value of risk-score. By analyzing the whole-exome data of CNV, it was found that compared with the low risk-score group, the high risk-score group had more characteristic chromosome amplification and deletion (**Figures 5C, D**), such as 8q, 9p, 17, 18, 19q, and 20q chromosome amplification, and 2q, 6, 7, and 8p chromosome deletion. At the same time, a total of 1,593 differential CNVs related to expression were generated, mainly enriched in immune-related functions, such as activation and positive regulation of immune response, participating in innate immune response and antigen receptor-mediated signaling pathway (**Figure 5E**) by GSEA, where there was a total of six intersection genes with DEGs generated by the transcriptome, that is, CA9, TNNT1, FABP4, CCL21, LY86, and CCL19 (**Figure 5F**).

### CCL19 Was a Potential Factor Affecting Risk-Score

**Figure 6:**
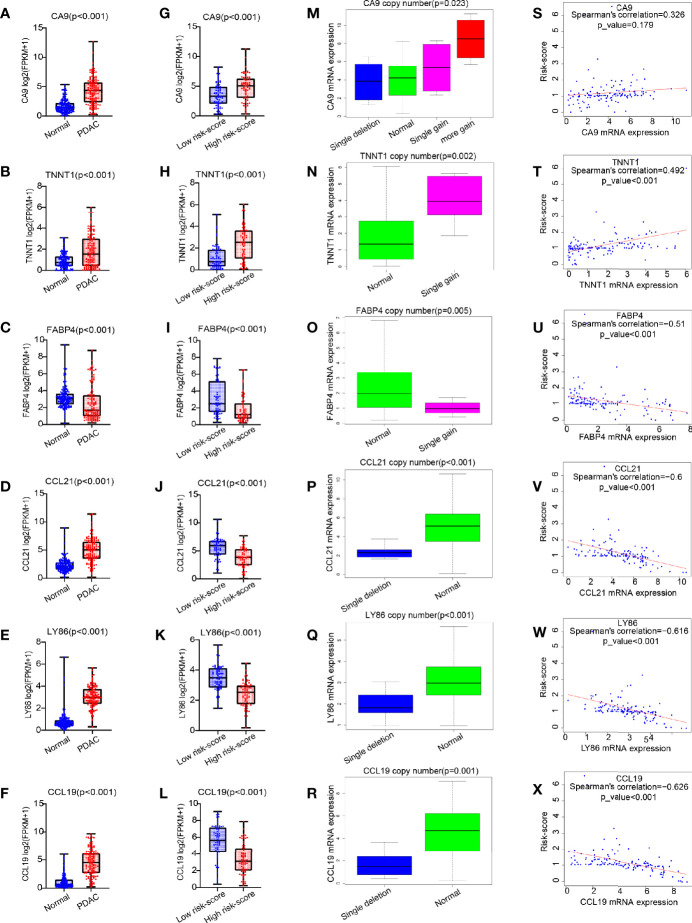
The expression pattern of six common genes and their correlation with risk score. **(A–L)** Box plot for analysis of six genes (CA9, TNNT1, FABP4, CCL21, LY86, and CCL19) expression difference in normal (TCGA + GTEx) *vs*. PDAC (TCGA), and high risk score *vs*. low risk score. **(M–R)** Box plot for analysis of the relationship between six gene expression (CA9, TNNT1, FABP4, CCL21, LY86, and CCL19) and CNV. **(S–X)** Correlation of six gene expression (CA9, TNNT1, FABP4, CCL21, LY86, and CCL19) with risk score. TCGA, The Cancer Genome Atlas; GTEx, Genotype-Tissue Expression; PDAC, pancreatic ductal adenocarcinoma; CNV, copy number variation.

Compared with those in the normal pancreas (GTEx + TCGA), CA9, TNNT1, CCL21, LY86, and CCL19 were all expressed higher in tumor tissues, except for FABP4, which was expressed lower in a tumor (p < 0.001, **Figures 6A**–**F**). In contrast with that in the low risk-score group, the expression of CA9 and TNNT1 increased in the high risk-score group, while FABP4, CCL21, LY86, and CCL19 were contrasting (p < 0.001, **Figures 6G**–**L**). Except for FABP4, their expression was positively correlated with the change of copy number. Unlike in the low risk-score group, in the high risk-score group, CA9 showed a variety of copy number changes; that is, in addition to the normal double copy number, there was also single copy number deletion, single copy number, or even multiple copy number amplification (p = 0.023, **Figure 6M**). TNNT1 and FABP4 mainly showed single copy number amplification (p = 0.002, p = 0.005, **Figures 6N, O**), while CCL21, LY86, and CCL19 mainly showed single copy deletion in the high risk-score group (p < 0.001, p < 0.001, and p = 0.001, **Figures 6P**–**R**). Furthermore, we analyzed the correlation between these six genes and risk-score, and we found that except for CA9 (**Figure 6S**), the other five genes were significantly correlated with risk-score. Among them, only the expression of TNNT1 was positively correlated with risk-score (R = 0.492, p < 0.001, **Figure 6T**), FABP4, CCL21, LY86, and CCL19 were negatively correlated with risk-score (R = −0.51, R = −0.6, R = −0.616, R = −0.626, p < 0.001, **Figures 6U**–**X**), while there was no statistical correlation between CA and risk-score.

**Figure 7:**
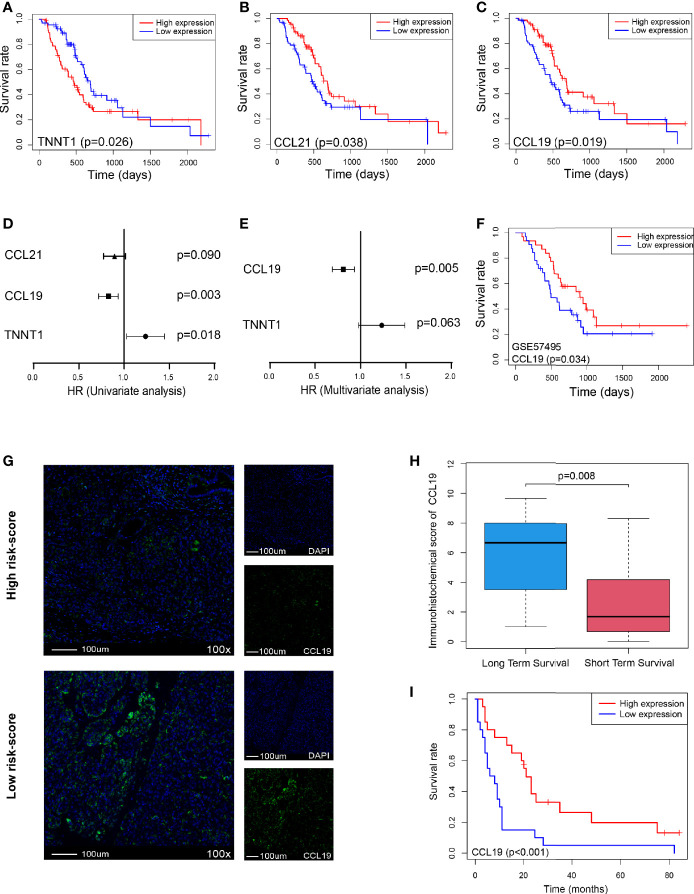
CCL19 is a potential factor affecting risk score. **(A–C)** OS in TNNT1, CCL21, and CCL19 high- *vs*. low-expression patients depicted by KM plots in TCGA PDAC discovery cohort, respectively. **(D, E)** Forest plot showing the HR of TNNT1, CCL21, and CCL19 in the univariate and multivariate Cox analyses, respectively, in TCGA PDAC discovery cohort. **(F)** OS in CCL19 high- *vs*. low-expression patients depicted by KM plots in GSE57495 validation cohort. **(G)** Multiplex immunofluorescence analyses on CCL19 expression in Peking2020 cohort (n = 40, randomly select three fields at least). **(H)** Box plot depicts CCL19 expression between long-term survival and short-term survival in Peking2020 cohort. **(I)** OS in CCL19 high- *vs*. low-expression patients depicted by KM plots in Peking2020 cohort. OS, overall survival; KM, Kaplan–Meier; HR, hazard ratios; TCGA, The Cancer Genome Atlas; PDAC, pancreatic ductal adenocarcinoma.

Furthermore, we analyzed the survival of these five genes and found that only TNNT1, CCL21, and CCL19 were closely related to survival, of which TNNT1 was negatively related to survival (p = 0.026, **Figure 7A**), while CCL21 (p = 0.038, **Figure 7B**) and CCL19 (p = 0.019, **Figure 7C**) were positively correlated with prognosis. Then univariate and multivariate analyses combined with clinical factors found that only CCL19 was positively correlated with patient survival (p = 0.003, **Figure 7D**) and could play a role as an independent prognostic factor (p = 0.005, **Figure 7E**). It was also verified in GSE57495 dataset concurrently (p = 0.034, **Figure 7F**). Similarly, in Peking2020 cohort, we also found that CCL19 expression was significantly lower in the high risk-score group (**Figure 7G**). Compared with the long-term-survival patients, CCL19 also showed a downward trend in the short-term-survival patients group (p = 0.008, **Figure 7H**). Further survival analysis showed that CCL19 was positively correlated with the prognosis (p < 0.001, **Figure 7I**), consistent with the above result, which suggested that CCL19 may be an important factor affecting CD4^+^T_N_ and CLP infiltration.

## Discussion

Using tumor immune microenvironment to assess tumor prognosis has attracted increasing attention, such as breast cancer, lung cancer, gastric cancer, and glioma. However, there was no consensus in PDAC. Unlike traditional prognostic evaluation model, we boldly and innovatively combined infiltrated immune cells to construct Cox model for prognostic evaluation in PDAC. In order to eliminate the bias caused by a single xCell algorithm, we used the TIMER and MCPCOUNTER algorithms to predict infiltrating immune cells. Due to the differences in the characteristic genes and calculation methods selected by different algorithms, they cannot accurately predict 36 different immune cells as finely as xCell, which impelled us to choose cell populations close to or containing CD4^+^T_N_ and/or CLP as much as possible. We found that the CD4^+^ T cells predicted by the TIMER algorithm and T cells predicted by MCPCOUNTER algorithm were positively correlated with the prognosis (**Supplementary Figures S1H, I**), which indirectly supported the reliability of our results by xCell algorithm. Furthermore, the feasibility of our model to predict prognosis has also been deeply verified in GEO datasets and our Peking2020 cohort. We found that the risk-score originated Cox model was not affected by the demographic characteristics of age, gender, and race; nor was it interfered by conventional high-risk factors for PDAC such as smoking, drinking, diabetes, chronic pancreatitis, and family history of tumors. Besides, there was no significant correlation between the TNM stage, tumor stage, surgical method, and risk-score (**Supplementary Figure S2**). At the same time, as for the commonly used bio-markers of PDAC, such as CA199, CA125, and carcinoembryonic antigen (CEA), there was no significant difference among them in the expression of tumor tissues between the high risk-score and low risk-score groups, which suggested that these traditional indicators to predict prognosis had a certain lag. Based on the above, the risk-score could be used as an independent prognostic factor. Nevertheless, the risk-score had nothing to do with the anatomical site of tumor occurrence in TCGA database, but tumor in the tail of the pancreas had a higher risk-score than the head of the pancreas in our Peking2020 cohort unexpectedly, where the reasons needed to be further explored. In addition, chemotherapy was closely related to the prognosis of patients. In our study, we found that chemotherapy can significantly improve the prognosis of patients, whether in the high risk-score or low risk-score group (**Supplementary Figures S3A**–**C**). There was no significant difference in risk-score among patients who received or did not receive chemotherapy (**Supplementary Figure S3D**). However, the high risk-score group always had a worse prognosis than the low risk-score group regardless of whether the patient received chemotherapy or not (**Supplementary Figures S3E, F**). Interestingly, our risk-score has higher prognostic prediction sensitivity in the patient without chemotherapy group than with chemotherapy group (without chemotherapy *vs*. with chemotherapy AUC (1 year) = 0.743 *vs*. 0.657, without chemotherapy *vs*. with chemotherapy AUC (3 years) = 0.782 *vs*. 565; **Supplementary Figures S3G, H**).

It was well known that PDAC was a “cold” tumor, although the TMB of the high risk-score group was higher than that of the low risk-score; based on the background of low TMB overall, this difference may not be sufficient to assess its sensitivity to immunotherapy. Fortunately, the risk-score still worked well to predict the sensitivity to immunotherapy, and the patients possessing higher cytotoxicity or using molecular targeted therapy all tended to have a lower risk-score. Unfortunately, due to the current lack of immunotherapy sensitivity indicators, PDAC patients seldom received immunotherapy. Therefore, although we can predict the sensitivity of immunotherapy, we failed to collect enough patients who had received immunotherapy, and the detailed impact of risk-score on the prognostic performance in immunotherapy cohort cannot be accurately assessed. The population must be further expanded, and more complete information must be collected for in-depth exploration. CNV played a fundamental role in tumorigenesis and development. It could be used not only as a prognostic indicator but also as a therapeutic target; for example, Herceptin and Iressa were developed for HER2 and EGFR copy number amplification, respectively. In our research, we analyzed the differences of CNV to explore the potential mechanism of why our model can evaluate the prognosis. Finally, CCL19 was found from 1,593 differential copy numbers that can be transcribed. Copy number deletion or low expression of CCL19 may be key to potential impact on risk-score.

Previous studies had shown that CCL19 (23, 24) was one of the most significant chemokines, produced by dendritic cells (DCs), lymphocytes, and some non-lymphocytes, including tumor cells, consistent with our results. Moreover, CCL19 could specifically bind to its receptor, chemokine receptor 7 (CCR7), a class A subtype 7-span transmembrane G-protein coupled receptor, which was expressed on DCs, natural killer (NK) cells, macrophages, and lymphocytes including CD4^+^T_N_ and CLP. Therefore, we speculated that PDAC may promote the infiltration of immune cells, especially CD4^+^T_N_ and CLP, by secreting CCL19. The effect of CD4^+^T_N_ on tumor prognosis has not been unified. Some research groups had pointed out that CD4^+^T_N_ often indicated poor prognosis of breast cancer (25). However, some scholars hold the view that CD4^+^T_N_ level in smoking lung cancer is correlated with favorable prognosis (26). These contradictory conclusions may depend on which kind of cells CD4^+^T_N_ was finally differentiated into. In our study, CD4^+^T_N_ supported favorable prognosis, also possibly because CD4^+^T_N_ differentiated into CD4^+^Th1 cells induced by the unique microenvironment of PDAC, which further enhanced CD8^+^T cell cytotoxicity synergistically and conditioned B cells to promote the secretion of corresponding antibodies in line with our prediction in GO annotation. Although CLP may differentiate into various types of lymphocytes, it may be induced by the unique microenvironment of PDAC, whose outcome was similar to that of common myeloid progenitor (CMP) differentiated into MDSC (27), and finally differentiated into lymphoid-derived suppressor cells, which is correlated with poor prognosis.

In addition, apart from CCL19, chemokines such as CCL2, CCL3, CCL4, CCL5, CCL8, CXCL13, CCL18, and CCL21 (**Supplementary Figure S4A**) in the low risk-score group also increased significantly, suggesting that there were more other immune cell infiltration. Although these were not essential factors affecting our model and the prognosis, at least they reflected that the overall immune status of the low risk-score group was much better than that of the high risk-score group, on the other hand. Simultaneously, in our study, it was also found that the low risk-score group had more expression of T cell-activated co-stimulatory factors (such as ICOSLG, CD40LG, TNFRSF9, TNFRSF4, TNFSF18, TNFRSF18, ICOS, CD28, CD27, and CD40) and co-inhibitory factors (such as CTLA4, PDCDL1G2, PDCD1, VSIR, LAG3, HAVCR2, and TIGIT) (**Supplementary Figures S4B, C**), suggesting that PDAC TME was not a simple binary state of activation or inactivation, which may be related to the asynchronous activation and inactivation of infiltrating immune cells. At the same time, each molecule was expressed continuously rather than in isolation during the process from activation to inactivation.

Although studies around PDAC traditional molecular subtype have paid attention to the indispensable role of TME and made great efforts to predict the prognosis of pancreatic cancer, there was a limitation for these findings in clinical transformation in that the genes applied to prediction had not yet reach consensus, as well as the need for complex algorithms and transcriptome sequencing to classify them. Instead, our model was mainly based on xCell and Cox algorithms; not only that the calculation was more concise, but also the high-risk molecular subtypes can be distinguished well. What is more, besides evaluation through transcriptome sequencing data, our model can also use economical multiplex immunofluorescence staining to achieve prognostic evaluation alternately. Additionally, previous studies had also suggested that high perineural invasion was correlated with poor prognosis of PDAC (28, 29), whereas high neural density tended to have a better prognosis (30). Consistent with the latter, in our model, the activation of nerve fibers mainly occurred in the low risk-score group by GSEA (**Supplementary Figures S5A**–**D**), which may drop a hint that more neuro-immune cell units (NICUs) (31), that is, the co-localization of nerve fibers with immune cells, may be formed in the low risk-score group. As it happened, NICUs and their interaction can inhibit tumor progression by driving tissue protection and immune regulation, which further supported the prominent position of our model from another dimension.

In future clinical work, we can also learn from this research idea by analyzing transcriptome sequencing data and then using the xCell algorithm to predict the level of CD4^+^T_N_ and CLP infiltration, or using multiplex immunofluorescence staining to evaluate the degree of CD4^+^T_N_ and CLP infiltration per ×100 magnification field and then using the algorithm of our model for reference to calculate the risk-score and establish a complete reference interval of prognostic evaluation scoring based on our model. However, due to the low detection rate of early PDAC, it was mostly in the advanced stage when the opportunity for surgery was lost, which was a huge obstacle for us in collecting the early- or late-stage samples. In TCGA PDAC datasets, there were only 11 cases with stage I and only three cases with stage IV in PDAC (136 cases in total). Similarly, there were only four cases with stage I and only one case with stage IV in our Peking2020 cohort (40 cases in total). This led to the lack of sufficient early- and late-stage data in our model for training and validation, which may limit the accuracy of our model in evaluating the prognosis of such patients and reduce the sensitivity to predict tumor stage to some degree. In the future, it was expected to further expand population data, especially the early- and late-stage patients, for verification and to promote the clinical application of our model.

## Data Availability Statement

The datasets presented in this study can be found in online repositories. The names of the repository/repositories and accession number(s) can be found in the article/**Supplementary Material**.

## Ethics Statement

The studies involving human participants were reviewed and approved by the Ethics Committee of the Cancer Institute (Hospital), CAMS, and PUMC (17-168/1424). The patients/participants provided their written informed consent to participate in this study.

## Author Contributions

Conception and design: JM and WY. Data collection and experiment: BY, MZ, YW, YM, and QT. Analysis and interpretation of data: BY and QT. Writing, review, and/or revision of the manuscript: JM, WY, QT, and BY. Administrative, technical, or material support: JM, WY, and QT. Study supervision: JM. All authors contributed to the article and approved the submitted version.

## Funding

This study was supported by the National Key Research and Development Program of China (#2016YFA0201503), National Natural Science Foundation of China (#51972343, 51937011, 82003264), Disciplines Construction Project (#201920202103), and Beijing Hospital Project (#BJ-2019-134).

## Conflict of Interest

The authors declare that the research was conducted in the absence of any commercial or financial relationships that could be construed as a potential conflict of interest.

## Publisher’s Note

All claims expressed in this article are solely those of the authors and do not necessarily represent those of their affiliated organizations, or those of the publisher, the editors and the reviewers. Any product that may be evaluated in this article, or claim that may be made by its manufacturer, is not guaranteed or endorsed by the publisher.

## Supplementary Material

Supplementary Figure S1
**(A, B)** OS in high CD4+Th2 cell *vs*. low CD4+Th2 cell patients in TCGA PDAC dataset, high risk-score *vs*. low risk-score patients in GSE71729 dataset depicted by KM plots respectively. **(C)** ROC curves to depict the accuracy of risk-score in identifying poor OS in GSE71729 dataset for 1-year. **(D, E)** Box plot to depic risk-score of tumor-dimension <=3 *vs*. >3 centimeter, anatomic site of tumorigenesis, tail *vs*. head of pancreas in Peking2020 cohort respectively. **(F, G)** PCA analysis among normal pancreatic tissue (including normal pancreatic tissue in GTEX and paracancerous in TCGA PDAC dataset), high risk-score and low risk-score, and **(G)** is the reanalysis of high risk-score and low risk-score in **(F)**. **(H, I)** OS in high CD4+T cell *vs*. low CD4+T cell patients predicted by TIMER, high T cell *vs*. low of T cell predicted by MCPCOUNTER in TCGA PDAC dataset depicted by KM plots respectively. KM, Kaplan Meier survival analysis; OS, Overall Survival; PCA, Principal Component Analysis.

Supplementary Figure S2 The relation between risk-score and demographic and clinical characteristics in the TCGA PDAC dataset. Box plot to analyse risk-score in different age group (<=65 years old *vs*. >65 years old), different gender group (female *vs*. male), different race group (Asian, Black/African American *vs*. White), different history of smoke group (none, occasional *vs*. frequent), different history of alcohol group (no *vs*. yes), different history of diabetes group (no *vs*. yes), different history of chronic pancreatitis group (no *vs*. yes), different prior malignancy diagnoses group (no *vs*. yes), different anatomic site of tumorigenesis group (body, head, tail *vs*. overlap/other site of pancreas), different pathologic_M group (M0 *vs*. M1), different pathologic_N group (N0 *vs*. N1), different pathologic_T group (T1-T2 *vs*. T3-T4), different tumor stage group (stage I, stage II *vs*. stage III-IV), different surgery performed type group (no whipple *vs* whipple), different family history of cancer group (no *vs*. yes), respectively. The pathologic TNM staging system was based on seventh edition of the American Joint Committee on Cancer staging system in TCGA PDAC dataset.

Supplementary Figure S3 Interaction between chemotherapy on risk-score in TCGA PDAC dataset. **(A–C)** OS in the patients with Chemotherapy *vs*. without Chemotherapy in all-patient, high risk-score group and low risk-score group from TCGA PDAC dataset depicted by KM plots respectively. **(D)** Violin plot for analysis risk-score value between patients with Chemotherapy and without Chemotherapy group in TCGA PDAC dataset. **(E–F)** OS in high risk-score *vs*. low risk-score patients with chemotherapy or without chemotherapy from TCGA PDAC dataset depicted by KM plots, respectively. **(G–H)** ROC curves to depict the accuracy of risk-score in identifying poor OS of patients with chemotherapy or without chemotherapy in TCGA PDAC discovery cohort for 1-year and 3-year, respectively. KM, Kaplan Meier survival analysis; Chemotherapy, the patients with chemotherapy, no Chemotherapy, the patients without chemotherapy; ns, no statistical significance.

Supplementary Figure S4 The relation between risk-score and chemokine **(A),** co-stimulatory factor **(B)**, co-inhibitory factor **(C)** in the TCGA PDAC dataset. **(A)** Box plot for analysis chemokine (CCL2, CCL3, CCL4, CCL5, CCL8, CCL18, CCL19, CCL21, CXCL9, CXCL10, CXCL11 and CXCL13) expression difference in high risk-score *vs*. low risk-score, respectively. **(B)** Box plot to depict co-stimulatory factor (CD28, CD27, CD27LG, CD40, CD40LG, ICOS, ICOSLG, TNFSF4, TNFRSF4, TNFSF9, TNFRSF9, TNFSF18 and TNFRSF18) expression difference in high risk-score *vs*. low risk-score, respectively. **(B)** Box plot to illustrate co-inhibitory factor (CTLA4, PDCD1LG1, PDCD1LG2, PDCD1, VSIR, LAG3, LAGLS9, HAVCR2, DIDO1 and TIGIT) expression difference in high risk-score *vs*. low risk-score, respectively. ns, no statistical significance.

Supplementary Figure S5 Differential CNV function annotation and prediction by GSEA enrichment analysis in TCGA PDAC dataset. **(A)** Bar chart showing the top 10 CC-GO terms of differential CNV for high risk-score *vs*. low risk-score. **(B, C)** GSEA-based GO analysis-enrichment plots of representative gene sets: axon part, neuron projection enrichment in low risk-score, respectively. **(D)** GSEA-based KEGG-enrichment plots of representative gene sets from activated pathway: neurotrohin signaling pathway in low risk-score. In the GSEA plots, the vertical axis represents enrichment score. The enrichment score increased with the number of enriched genes and vice versa. CNV, copy-number variation; GSEA, Gene Set Enrichment Analysis; ES, enrichment score; NES, normalized enrichment score; GO, Gene Ontology; CC, Cellular Component; KEGG, Kyoto Encyclopedia of Genes and Genomes.

## Glossary

**Table:**

APLNR	Apelin receptor
CA9	Carbonic anhydrase 9
CCL18	C-C motif chemokine ligand 18
CCL19	C-C motif chemokine ligand 19
CCL2	C-C motif chemokine ligand 2
CCL21	C-C motif chemokine ligand 21
CCL3	C-C motif chemokine ligand 3
CCL4	C-C motif chemokine ligand 4
CCL5	C-C motif chemokine ligand 5
CCL8	C-C motif chemokine ligand 8
CCR7	C-C motif chemokine receptor 7
CD19	CD19 molecule
CD2	CD2 molecule
CD22	CD22 molecule
CD27	CD27 molecule
CD27LG	CD70 molecule/CD27 ligand
CD28	CD28 molecule
CD40	CD40 molecule
CD40LG	CD40 ligand
CD79A	CD79a molecule
CR2	Complement C3d receptor 2
CTLA4	Cytotoxic T-lymphocyte associated protein 4
CXCL10	C-X-C motif chemokine ligand 10
CXCL11	C-X-C motif chemokine ligand 11
CXCL12	C-X-C motif chemokine ligand 12
CXCL13	C-X-C motif chemokine ligand 13
CXCL9	C-X-C motif chemokine ligand 9
CXCR4	C-X-C motif chemokine receptor 4
DIDO1	Death inducer-obliterator 1
FABP4	Fatty acid binding protein 4
HAVCR2	Hepatitis A virus cellular receptor 2
ICOS	Inducible T cell costimulator
ICOSLG	Inducible T cell costimulator ligand
IGLL5	Immunoglobulin lambda like polypeptide 5
LAG3	Lymphocyte activating 3
LGALS9	Galectin 9
LY86	Lymphocyte antigen 86
MS4A1	Membrane spanning 4-domains A1
PDCD1	Programmed cell death 1
PDCD1LG1	Programmed cell death 1 ligand 1
PDCD1LG2	Programmed cell death 1 ligand 2
SAA1	Serum amyloid A1
SELL	Selectin L
SST	Somatostatin
TIGIT	T cell immunoreceptor with Ig and ITIM domains
TNFRSF18	TNF receptor superfamily member 18
TNFRSF4	TNF receptor superfamily member 4
TNFRSF9	TNF receptor superfamily member 9/4-1BB
TNFSF18	TNF superfamily member 18
TNFSF4	TNF superfamily member 4
TNFSF9	TNF superfamily member 9/4-1BB ligand
TNNT1	Troponin T1, slow skeletal type
VSIR	V-set immunoregulatory receptor
