# Supplementary figures and images for: The Impact of Immune Microenvironment on the Prognosis of Pancreatic Ductal Adenocarcinoma Based on Multi-Omics Analysis
Source: Front Immunol. 2021 Oct 28;12:769047. doi: 10.3389/fimmu.2021.769047 (PMC8580856; doi:10.3389/fimmu.2021.769047)

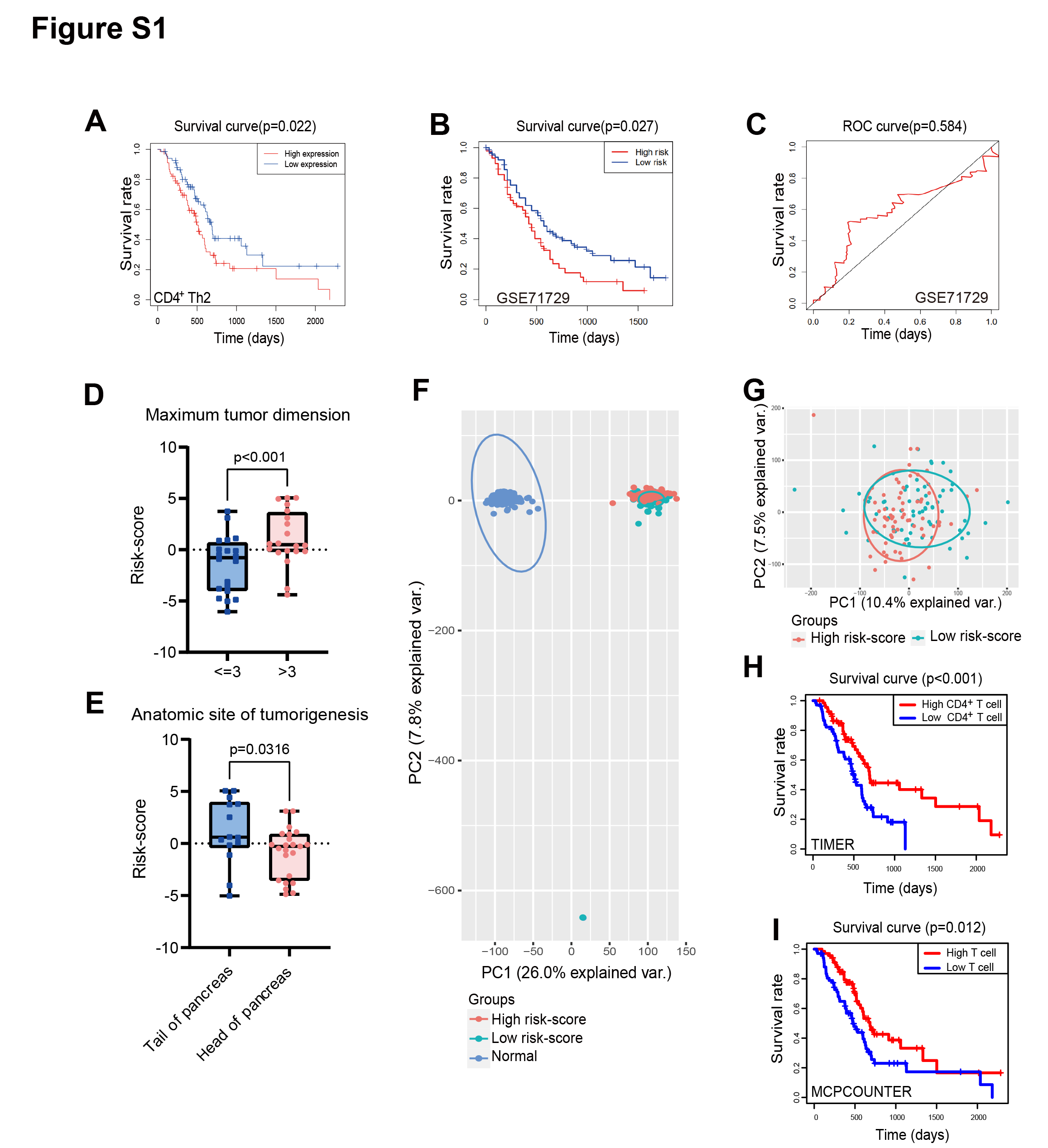

Supplement: Supplementary Figure S1 — (A, B) OS in high CD4+Th2 cell vs. low CD4+Th2 cell patients in TCGA PDAC dataset, high risk-score vs. low risk-score patients in GSE71729 dataset depicted by KM plots respectively. (C) ROC curves to depict the accuracy of risk-score in identifying poor OS in GSE71729 dataset for 1-year. (D, E) Box plot to depic risk-score of tumor-dimension <=3 vs. >3 centimeter, anatomic site of tumorigenesis, tail vs. head of pancreas in Peking2020 cohort respectively. (F, G) PCA analysis among normal pancreatic tissue (including normal pancreatic tissue in GTEX and paracancerous in TCGA PDAC dataset), high risk-score and low risk-score, and (G) is the reanalysis of high risk-score and low risk-score in (F). (H, I) OS in high CD4+T cell vs. low CD4+T cell patients predicted by TIMER, high T cell vs. low of T cell predicted by MCPCOUNTER in TCGA PDAC dataset depicted by KM plots respectively. KM, Kaplan Meier survival analysis; OS, Overall Survival; PCA, Principal Component Analysis. [file DataSheet_1.zip › Figure S1.tif]

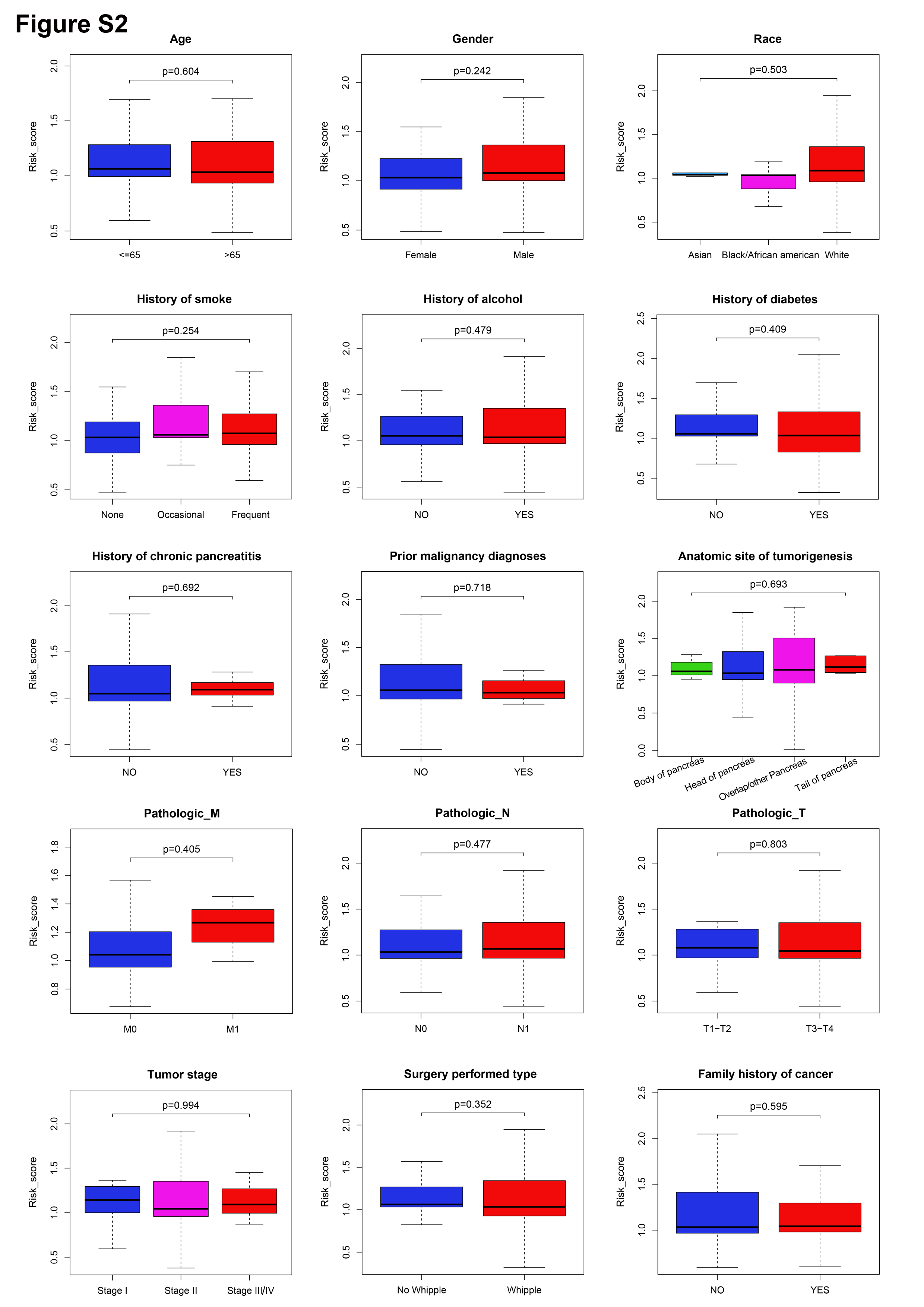

Supplement: Supplementary Figure S1 — (A, B) OS in high CD4+Th2 cell vs. low CD4+Th2 cell patients in TCGA PDAC dataset, high risk-score vs. low risk-score patients in GSE71729 dataset depicted by KM plots respectively. (C) ROC curves to depict the accuracy of risk-score in identifying poor OS in GSE71729 dataset for 1-year. (D, E) Box plot to depic risk-score of tumor-dimension <=3 vs. >3 centimeter, anatomic site of tumorigenesis, tail vs. head of pancreas in Peking2020 cohort respectively. (F, G) PCA analysis among normal pancreatic tissue (including normal pancreatic tissue in GTEX and paracancerous in TCGA PDAC dataset), high risk-score and low risk-score, and (G) is the reanalysis of high risk-score and low risk-score in (F). (H, I) OS in high CD4+T cell vs. low CD4+T cell patients predicted by TIMER, high T cell vs. low of T cell predicted by MCPCOUNTER in TCGA PDAC dataset depicted by KM plots respectively. KM, Kaplan Meier survival analysis; OS, Overall Survival; PCA, Principal Component Analysis. [file DataSheet_1.zip › Figure S2.tif]

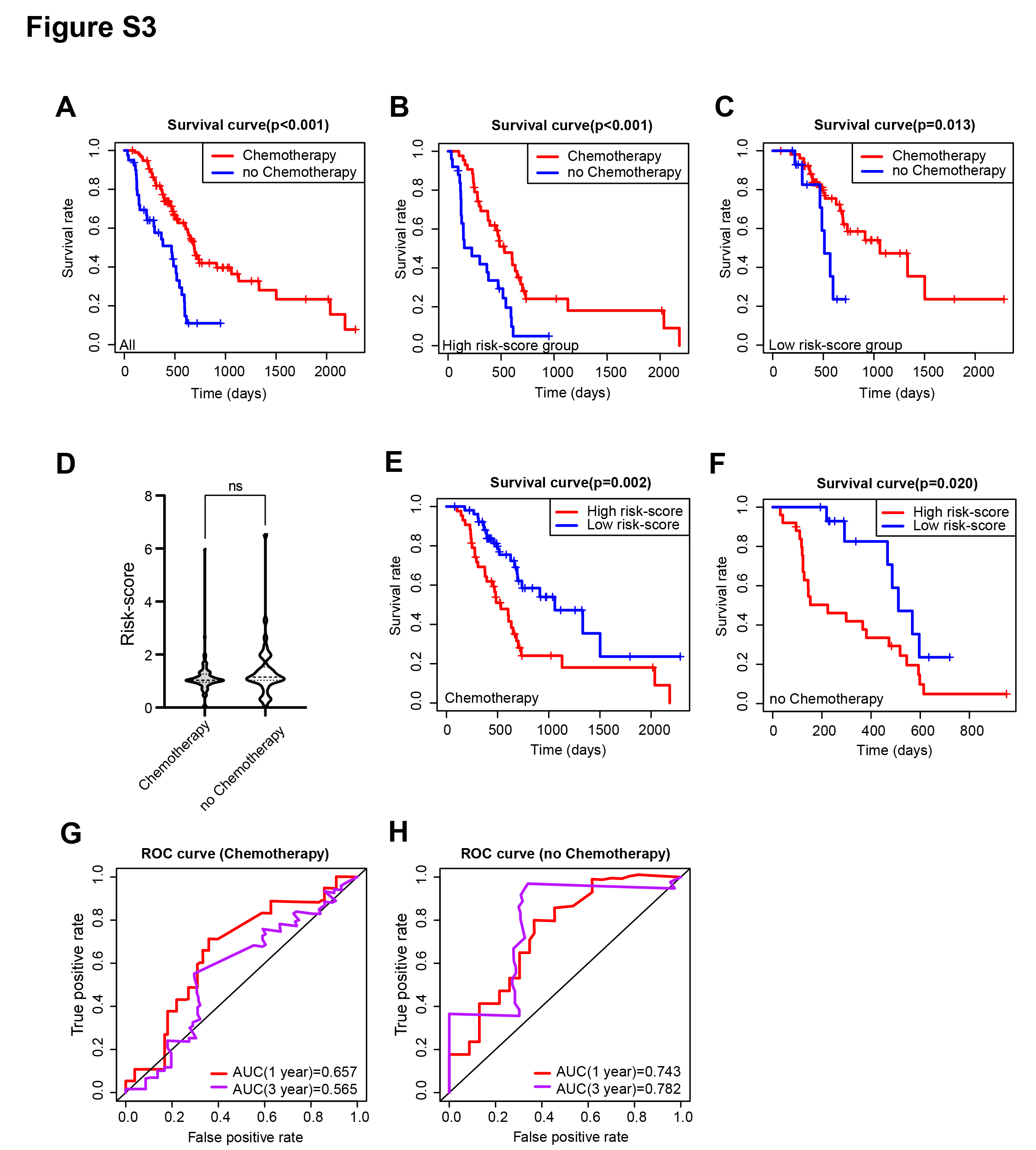

Supplement: Supplementary Figure S1 — (A, B) OS in high CD4+Th2 cell vs. low CD4+Th2 cell patients in TCGA PDAC dataset, high risk-score vs. low risk-score patients in GSE71729 dataset depicted by KM plots respectively. (C) ROC curves to depict the accuracy of risk-score in identifying poor OS in GSE71729 dataset for 1-year. (D, E) Box plot to depic risk-score of tumor-dimension <=3 vs. >3 centimeter, anatomic site of tumorigenesis, tail vs. head of pancreas in Peking2020 cohort respectively. (F, G) PCA analysis among normal pancreatic tissue (including normal pancreatic tissue in GTEX and paracancerous in TCGA PDAC dataset), high risk-score and low risk-score, and (G) is the reanalysis of high risk-score and low risk-score in (F). (H, I) OS in high CD4+T cell vs. low CD4+T cell patients predicted by TIMER, high T cell vs. low of T cell predicted by MCPCOUNTER in TCGA PDAC dataset depicted by KM plots respectively. KM, Kaplan Meier survival analysis; OS, Overall Survival; PCA, Principal Component Analysis. [file DataSheet_1.zip › Figure S3.tif]

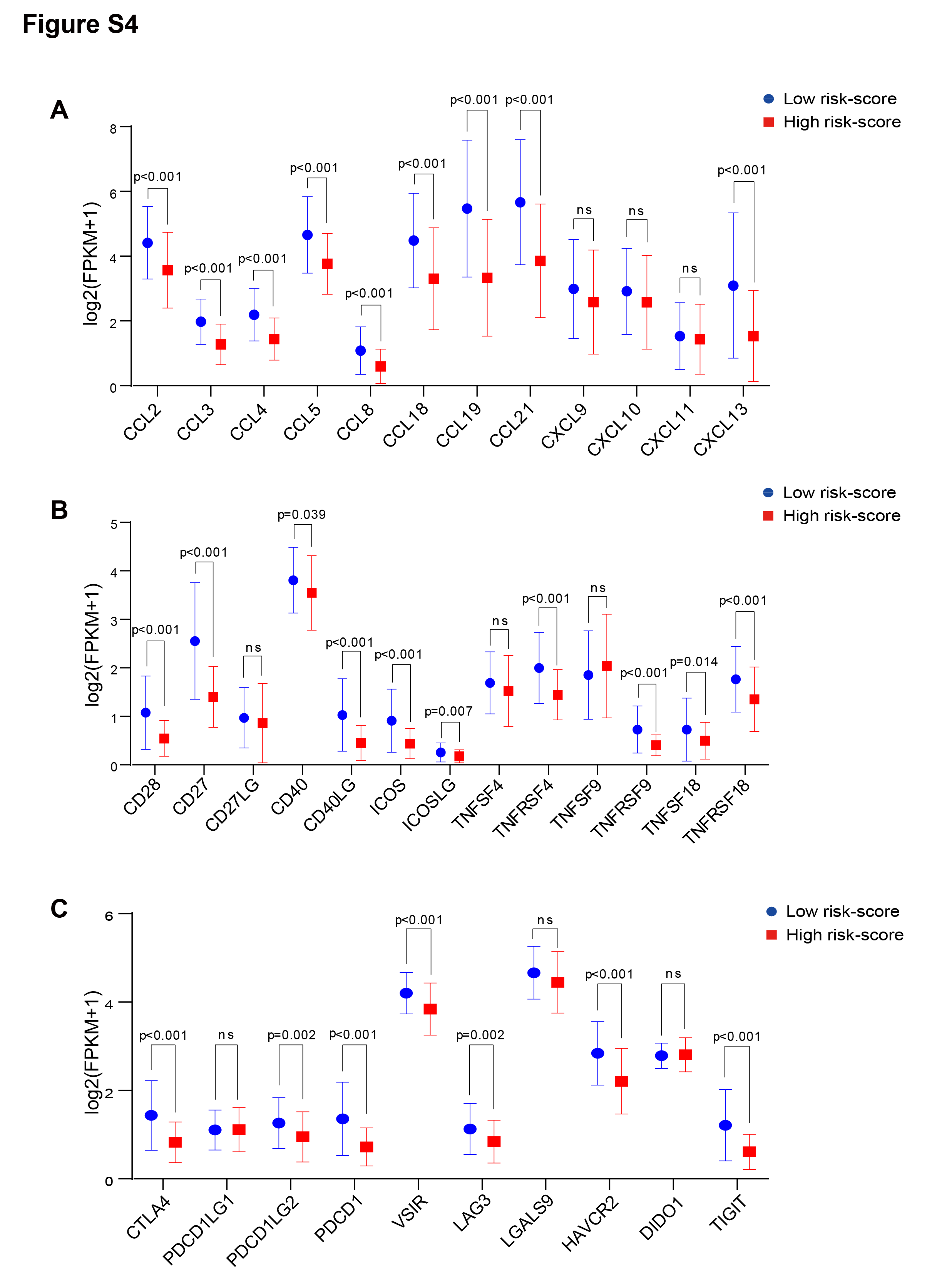

Supplement: Supplementary Figure S1 — (A, B) OS in high CD4+Th2 cell vs. low CD4+Th2 cell patients in TCGA PDAC dataset, high risk-score vs. low risk-score patients in GSE71729 dataset depicted by KM plots respectively. (C) ROC curves to depict the accuracy of risk-score in identifying poor OS in GSE71729 dataset for 1-year. (D, E) Box plot to depic risk-score of tumor-dimension <=3 vs. >3 centimeter, anatomic site of tumorigenesis, tail vs. head of pancreas in Peking2020 cohort respectively. (F, G) PCA analysis among normal pancreatic tissue (including normal pancreatic tissue in GTEX and paracancerous in TCGA PDAC dataset), high risk-score and low risk-score, and (G) is the reanalysis of high risk-score and low risk-score in (F). (H, I) OS in high CD4+T cell vs. low CD4+T cell patients predicted by TIMER, high T cell vs. low of T cell predicted by MCPCOUNTER in TCGA PDAC dataset depicted by KM plots respectively. KM, Kaplan Meier survival analysis; OS, Overall Survival; PCA, Principal Component Analysis. [file DataSheet_1.zip › Figure S4.tif]

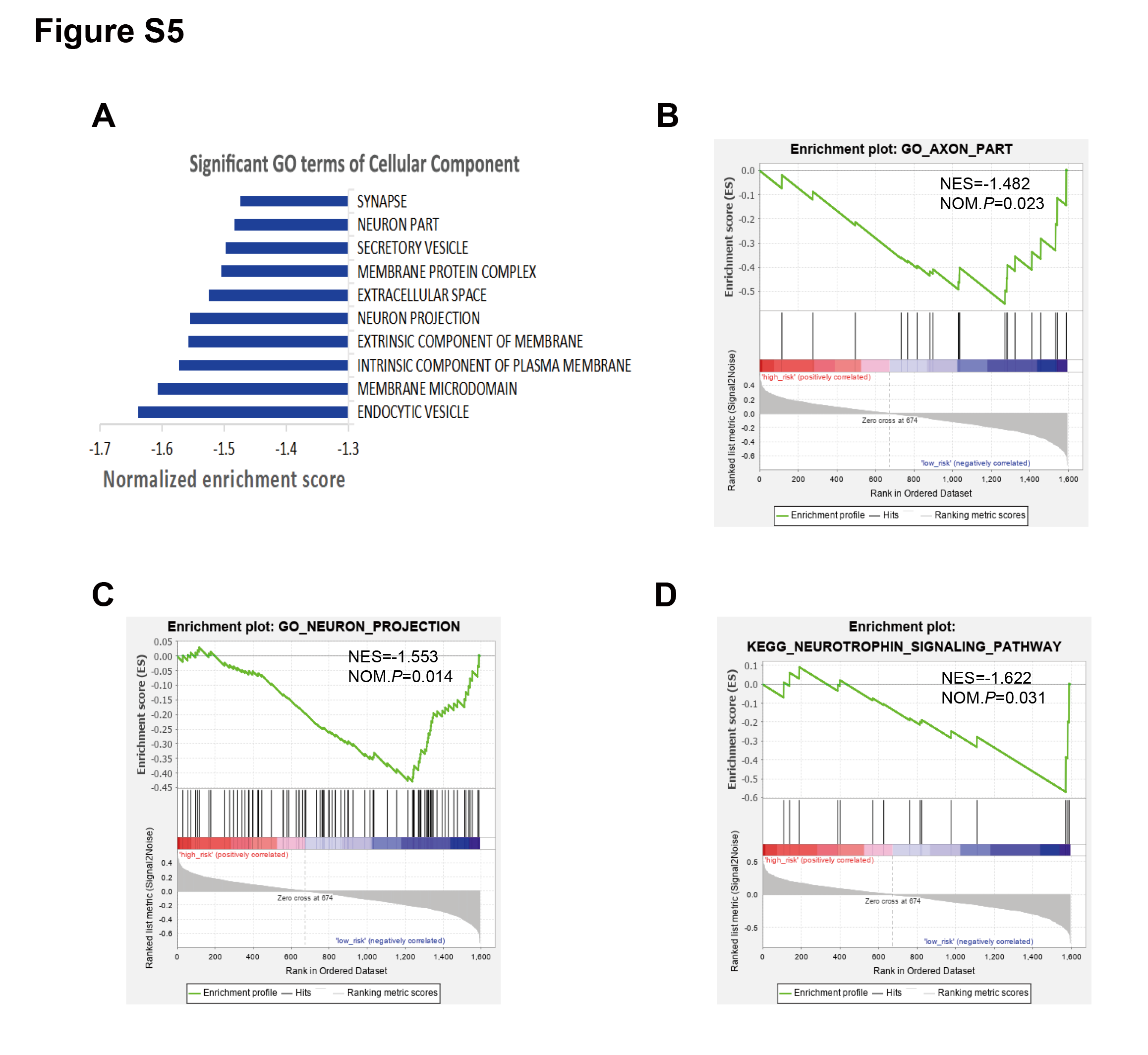

Supplement: Supplementary Figure S1 — (A, B) OS in high CD4+Th2 cell vs. low CD4+Th2 cell patients in TCGA PDAC dataset, high risk-score vs. low risk-score patients in GSE71729 dataset depicted by KM plots respectively. (C) ROC curves to depict the accuracy of risk-score in identifying poor OS in GSE71729 dataset for 1-year. (D, E) Box plot to depic risk-score of tumor-dimension <=3 vs. >3 centimeter, anatomic site of tumorigenesis, tail vs. head of pancreas in Peking2020 cohort respectively. (F, G) PCA analysis among normal pancreatic tissue (including normal pancreatic tissue in GTEX and paracancerous in TCGA PDAC dataset), high risk-score and low risk-score, and (G) is the reanalysis of high risk-score and low risk-score in (F). (H, I) OS in high CD4+T cell vs. low CD4+T cell patients predicted by TIMER, high T cell vs. low of T cell predicted by MCPCOUNTER in TCGA PDAC dataset depicted by KM plots respectively. KM, Kaplan Meier survival analysis; OS, Overall Survival; PCA, Principal Component Analysis. [file DataSheet_1.zip › Figure S5.tif]
